# Impending Upper Arm Compartment Syndrome Secondary to Intravenous Fluid Infiltration

**DOI:** 10.7759/cureus.15671

**Published:** 2021-06-15

**Authors:** Amr Tawfik, Bryan Hozack, Justin Melendez, Bobby Varghese, Brian M Katt, Pedro Beredjiklian, Michael Nakashian

**Affiliations:** 1 Division of Hand Surgery, Rothman Orthopaedic Institute, Philadelphia, USA; 2 Department of Orthopaedic Surgery, Robert Wood Johnson Medical School, New Brunswick, USA; 3 Division of Hand Surgery, Brielle Orthopedics, Brick, USA

**Keywords:** compartment syndrome, fasciotomy, radial nerve palsy, upper extremity, case report

## Abstract

We report the case of an 81-year-old female who developed an upper arm anterior compartment syndrome from the mass effect caused by an infiltrated intravenous access catheter. The patient’s anterior compartment became tense and uncompressible, and the patient developed radial nerve palsy. A fasciotomy was performed, resulting in the evacuation of 100 mL of fluid. Over the course of the patient’s follow-up, motor and sensory function slowly returned. In atraumatic patients with intravenous access, the development of a tense compartment with developing nerve palsies should warrant workup for possible compartment syndrome due to mass effect. If treated promptly with fasciotomy, the complications of this limb-threatening condition can be minimized or possibly reversed.

## Introduction

Compartment syndrome was initially described in 1881 by Volkmann when prolonged muscle ischemia was found to cause myonecrosis and secondary contracture [1]. It is now understood that progressive tissue ischemia occurs when arterial perfusion is diminished because of increased interstitial tissue pressure that constricts or collapses the arterial inflow [2].

The increase in compartment pressure can be intrinsic or extrinsic in etiology. Intrinsic causes can be the result of soft tissue swelling or intra-compartmental bleeding after an injury. The most common causes of compartment syndrome are related to a traumatic injury such as a fracture or dislocation [2]. Extrinsic causes include tightly wrapped casts or splints, prolonged tourniquet use, and external compression from prolonged surgical positioning [3]. The risk of compartment syndrome is increased with bleeding disorders or anticoagulation therapy, volume resuscitation, altered mental status, neurologic compromise, or post-viral rhabdomyolysis [4,5]. Iatrogenic injuries have been reported to occur after radial arterial puncture, intraosseous fluid resuscitation, longitudinal traction used in fracture reduction, improper elevation of a limb, and reperfusion injury after prolonged ischemia [4,6].

While compartment syndrome of the upper limb most commonly occurs within the hand or forearm, it can rarely develop in the upper arm [3,7,8]. Here, we present the case of an 81-year-old female with the signs and symptoms of an upper arm anterior compartment syndrome following a collection of intravenous fluid from an infiltrated intravenous access catheter. The patient was informed that data from the case would be submitted for publication, and she gave her consent.

## Case presentation

An 81-year-old female patient was admitted through the emergency department for abdominal pain and suspected small bowel obstruction. Intravenous access was established through an antecubital catheter in the right upper extremity. Additionally, the patient was placed on a heparin drip with access through the right antecubital fossa for atrial fibrillation. On the second day after admission, she complained of significant pain and swelling in the right upper arm. It was noted the intravenous catheter placed in the antecubital fossa had infiltrated, so it was subsequently removed. The patient’s pain progressed, and she began to develop numbness and tingling in the right hand, accompanied by a wrist drop. There were no signs of bruising or other evidence of any previous injury to the area, and the patient did not have pain in her shoulder or neck. There were no signs of extrinsic compression from the bedrail, blood pressure cuff, or the patient's clothing. An orthopedic consultation was called to evaluate for possible compartment syndrome. The patient was lethargic following Ativan administration but was able to comply with the examination. The upper arm anterior compartment was tense and not compressible, with pain reproducible with palpation. A passive stretch of the biceps induced significant pain. She was unable to extend her right wrist or fingers against gravity and had diminished light touch sensation over the dorsal and volar surfaces of the right hand. Wrist flexion was intact, the posterior compartment and forearm were soft and compressible, and a 2+ radial pulse was palpable. Based on the clinical diagnosis of impending anterior arm compartment syndrome, the patient was taken emergently to the operating room. Compartment pressures were not measured preoperatively as the senior author was called in to assist the on-call orthopedic surgeon who had already prepped, draped, and made skin incision upon arrival into the operating room. 

Due to the predominance of elevated pressure in the anterior compartment and the presence of suspected radial nerve palsy, a lateral incision was made so that the radial nerve could be identified and decompressed as needed [9]. Careful hemostasis was maintained with electrocautery. The radial nerve was identified and was found to have no obvious defects. The deep brachial fascia over the anterior compartment was found to be tense. An approximately 30 cm long incision was made with subcutaneous undermining distally and proximally to obtain a full-length fasciotomy. The incision was made laterally at the level of the intermuscular septum, and the nerve was identified anterior to the septum. This resulted in bulging of the biceps muscle belly, although the muscle tissue appeared completely normal. Dissecting more medially, we encountered a jet propulsion of clear fluid that burst from the anterior compartment (Video 1). Approximately 100mL of intravenous fluid was identified isolated within a pocket between the brachialis and the long head of the biceps, which was then rapidly evacuated, decompressing the entire upper arm. The muscle bellies did not have any edematous tissue, and the soft tissues of the arm felt to be back to their regular consistency at this point. As the pressure was relieved and the radial nerve had no obvious defects, extensive neurolysis was not found to be necessary. Since the muscle bellies, fascial layers, and skin were no longer under any pressure, the skin was easy to reapproximate without tension, and primary skin closure was performed. Compartment pressures were not remeasured due to the complete relief of tension exhibited. The patient was discharged in a wrist splint.

**Video 1:** Fluid Extravasation After Fasciotomy

Up to one month postoperatively, the patient was found to have minor swelling of the anteromedial aspect of the right arm, with all compartments soft and compressible. On motor examination of the right side, she displayed 0/5 finger and wrist extension, 1/5 wrist flexion, 2/5 flexor pollicis longus (FPL) and flexor digitorum profundus (FDP) flexion, and 5/5 biceps and triceps activity. Her sensation was diminished to light touch over the dorsal and volar surfaces of the right hand. A follow-up electromyography (EMG) one and a half months postoperatively revealed absent voluntary motor unit recruitment in the right pronator teres, brachioradialis, first dorsal interossei, and abductor pollicis brevis. Her right flexor carpi ulnaris, paraspinal, and proximal muscles were found to be normal. Nerve conduction studies revealed abnormal motor responses in the right median and ulnar nerves and abnormal sensory responses in the right ulnar, median, and radial nerves. At her two-month follow-up, she was noted to have absent pain and improving sensation, with a slight reduction to light touch over the dorsum of the hand and the first two digits on the volar surface. On motor examination, her FPL and FDP flexion improved to 3/5, but the rest of the examination was unchanged. Five months postoperatively, she was noted to have a return of light touch sensation over the volar surface. On motor examination, her wrists and fingers were now able to flex to 45° passively, and motor strength improved to 5/5 flexion in the wrist, FDP, FPL, and flexor digitorum superficialis. This suggested that her median and ulnar nerve palsies were resolved, but her radial nerve palsy had persisted. The patient returned to the office 14 months postoperatively for an unrelated complaint of metacarpophalangeal joint osteoarthritis. Her neurologic function was retested, and she displayed 5/5 flexion and extension in the wrist, FDP, and FPL suggesting total motor recovery. She complained of slight paresthesias in the right forearm and hand but displayed intact light touch sensation in all nerve distributions.

## Discussion

Although the forearm and hand are more common sites for compartment syndrome in the upper extremity, the upper arm can be affected. In these cases, the cause of the compartment syndrome is most commonly a result of crush injuries or fractures of the humerus [2,10]. Soft tissue trauma, including bicep tendon rupture, minor contusion, and vigorous exercise, has been associated with the occurrence of upper arm compartment syndrome [7,8,11-13].

There have also been a few case reports of atraumatic causes of upper arm compartment syndrome. In one case, a patient underwent a twelve and a half hour mass excision surgery utilizing a tourniquet for hemostasis. Twelve hours postoperatively, the patient developed compartment syndrome requiring fasciotomy over the region of the tourniquet placement, with anterior compartment pressure of 70 mm Hg [3]. One case involved a patient who developed compartment syndrome due to IV fluid extravasation; however, the patient was heavily anesthetized, masking the early signs of compartment syndrome [14]. 

The lack of edematous tissues within the muscle bellies during the time of decompression makes our presented case unusual for compartment syndrome. Instead, a distinct pocket of intravenous fluid was identified at the time of surgical intervention, which remained discretely contained between the fascial planes. Typically, signs of reversible muscle damage can be seen with four hours of ischemia, but it can take around eight hours for irreversible damage to the muscles to occur [15]. It is likely that surgical intervention in our case was performed quickly enough to avoid noticeable muscle necrosis.

We were unable to find a case similar to ours with a collection of crystalloids requiring urgent decompression. However, this complication can occur due to the pressure gradient generated by pushing fluids into the body. Often when an IV becomes infiltrated, the fluid seeps into the adjacent soft tissues. This is accompanied by localized irritation and discomfort. Typically, this discomfort will prompt a patient to notify medical personnel to stop the infiltration. The site is then treated with warm compresses and analgesics for pain control. Therefore, it is likely that patients with barriers to communication (due to sedation or otherwise) or experiencing sensory impairment who are receiving IV therapy on a pressured system are at a higher risk of developing compartment syndrome [16].

Many mechanical pumps have a sensor to stop the delivery of fluid if an infiltration has occurred. It is unclear in our case if a localized pressure increase was not seen due to a malfunctioning sensor or the fluid tracking away from the IV site. Positioning the pump (and sensor) above the patient leads to a lower pressure measurement at the sensor than at the catheter tip. Thus, the pressure at the patient’s vein may be significantly higher than that sensed [17]. Additionally, an Australian study examined multiple infusion systems, finding that pressure monitoring pumps produced line pressures during occlusion that were consistently up to two times greater than alarm settings. Following an occlusion, line pressures remained 25 to 75 mm Hg higher than alarm pressures [18].

Weber et al. postulated additional causes of extravasation of IV-administered contrast material included vessel perforation caused by the contrast jet exiting the catheter, puncturing the back wall of the vein at the time of catheter insertion or multiple punctures of the wall at the time of catheter insertion due to difficulty in placement [19]. Any of these scenarios could have been possible in our case. If a deep vein was used for access, the indwelling catheter might have been placed deep into the anterior upper arm fascia. In another plausible scenario, the collection could have started superficial to the fascia and grew large enough to break through the anterior fascia. The fluid could have then collected further up the arm along fascial planes between two muscles.

For the baseline catheter with an end hole only, the maximum velocity occurs at the tip, which results in higher maximum vessel wall shear stress when the tip is pointed obliquely toward the vessel wall, potentially rupturing the vein and leading to infiltration [19]. With small catheters, as are often used when there is difficulty with insertion, the velocity at which the fluid is expelled increases as the lumen is decreased. This, again, can lead to perforation. In our case, a 20-gauge catheter was used, and the fluid rate was 74 mL/hr. It seems plausible that this pressure could cause venous damage and infiltration.

In most cases of compartment syndrome, primary wound closure is delayed, as the edematous tissue may make it impossible to perform primary closure or lead to recurrent compartment syndrome [20]. However, this can lead to complications, such as wound infection, delayed healing, and permanent muscle weakness [20]. In our case, there was no pressure in the muscle bellies, fascial layers, or skin, and there was no evidence of muscle edema. This allowed for the skin to be reapproximated without tension, so the senior author comfortably attempted primary closure.

## Conclusions

Upper arm compartment syndrome is uncommon but can be limb-threatening if not identified and treated expeditiously. In the current literature, most cases of upper arm compartment syndrome are related to a traumatic event. In hospitalized patients that develop a clinical examination consistent with compartment syndrome in the upper arm, the possibility of IV infiltration causing mass effect due to fluid collection should be considered. In such a case, it may not be necessary to leave the surgical wound open as the skin may be closed safely.
